# Genitourinary and Syphilitic Diseases

**Published:** 1902-10

**Authors:** Byron H. Daggett

**Affiliations:** Buffalo, N. Y.


### Genitourinary and Syphilitic Diseases.

Conducted by BYRON H. DAGGETT, M. D., Buffalo, N. Y.

### STONE IN THE KIDNEY.

A. H. Cordier (*International Journal of Surgery*, Nov., 1902,) states that it is some twenty years since Henry Morris, of London, deliberately and successfully removed a stone from the kidney. The fear of hemorrhage stayed the progress of kidney surgery for many years. When it was demonstrated that the fear was groundless, kidney surgery took rank among the justifiable and legitimate surgical procedures. The result has been a notable epoch in surgical history and the advance in kidney surgery has been unexcelled in any other department. The surgery of the kidney should be prophylactic and its purpose to prevent the destruction of the function of the organ.

Early diagnosis and early operation are called for in all surgical affections of the kidney. The advance of kidney surgery has demonstrated that nephritic calculi are more frequent than was previously thought and that lumbago, neuralgias and perinephritic abscess were often the results of nephritic calculi.

Urinary fistula will become less frequent when nephrotomy is done early in the case.

A wound in the healthy kidney will heal as readily as one in any other organ, so that the surgeon is justified in making an exploratory nephrotomy. Pyonephrosis and hydronephrosis are late manifestations of stone in the kidney and should be anticipated by an early operation. A stone in the kidney is a menace to life and to the function of not only the affected organ, but also its fellow, as calculous anuria is often sympathetically developed.

Stone may form in the kidney at any age and its composition varies according to the age of the patient; in children it consists of urate of ammonia, in adults of uric acid, while the phosphatic and oxalate of lime calculi are common in patients past 40 years of age.

These stones may vary in number from one to a hundred, and in size from a grain to several ounces, their shape differing according to size, location and composition.

It may be that several years elapse in the development of a stone of any magnitude, yet a phosphatic calculous may form very rapidly. A stone too large to pass through the ureter will develop symptoms indicating its presence. If small it may pass through the ureter without producing any manifestations of its existence. Such a calculous may pass the urethra or lodge in the bladder and act as a nucleus for a cystic calculus.

If the stone lodges in the ureter, it causes intense pain, retraction of the testicle, irritable bladder, transient anuria, followed by polyuria and blood, after its passage. Atypical cases demand study, diagnosis and relief.

If the stone is held in the pelvis of the kidney a characteristic group of symptoms are presented. Nephritic colic indicates the passage of a calculus through the ureter and this may be followed by a quiescent period of months or possibly years without recurrence. Hemorrhage and pain may be caused by exercise or jolting of the body. *Kidney* pain is located in the region of the kidney. A calculus may intermittingly block the ureter and cause intermitting hydronephosis; if infected, pyonephrosis. Permanent obstruction makes the condition continuous. Extensive infection of the pelvis causes the characteristic enlargement, and if the ureter be patent, abundant amount of pus may be found in the urine.

Cystitis is a permanent symptom of calculous pyuria. A calculus in the kidney always threatens functional safety.

In cases of large stones and suppuration, diagnosis is easy. A history of renal colic, lumbar pains on one side, uric acid and oxalate of lime crystals in the urine, tenderness, recurrent hematuria warrant a sufficiency of diagnosis to justify an exploratory incision. This procedure is safe and harmless.

---

### Alkaline Saliva.

Saliva freighted with germs being constantly swallowed, may produce fermentation in the stomach, which results in eructations, sour stomach, headaches. Clinical experience shows that a small portion of a suitable alkaline solution will quickly correct the disorder. If the saliva is kept in its normally alkaline state, or in excess of normal, it seems to assist digestion instead of interfering with it.

The physiology of the mouth and stomach are so related that a remedy which will arrest putrefaction and fermentation in one organ, may do so in another and mild alkalies are most acceptable for this purpose.

The saliva must be utilised for disseminating an antiseptic and antacid, so that the cavities of decay will be penetrated by it.

Something must be used which will act upon mucous membrane for the purpose of keeping it in health or restoring it when it shows signs of disorder. The ingredients of such a preparation should be agreeable to each other and acceptable to the stomach, for the saliva passes in almost a constant stream to this organ, a fact too little considered in the treatment of the mouth.

Alkaline saliva seems an undoubted aid to digestion and if it can be induced to flow and be kept alkaline, many stomach disorders will disappear. The mucous membrane under the action of glyco-thymoline (Kress) becomes hardened and normal, and naturally offers greater resistance to disease; the daily application of the remedy as a mouth wash does much good, maintaining an alkaline or normal condition.—*Clinics.*

